# Batch production of 6-inch uniform monolayer molybdenum disulfide catalyzed by sodium in glass

**DOI:** 10.1038/s41467-018-03388-5

**Published:** 2018-03-07

**Authors:** Pengfei Yang, Xiaolong Zou, Zhepeng Zhang, Min Hong, Jianping Shi, Shulin Chen, Jiapei Shu, Liyun Zhao, Shaolong Jiang, Xiebo Zhou, Yahuan Huan, Chunyu Xie, Peng Gao, Qing Chen, Qing Zhang, Zhongfan Liu, Yanfeng Zhang

**Affiliations:** 1 Department of Materials Science and Engineering, College of Engineering, Peking University, Beijing, 100871 China; 2 Center for Nanochemistry (CNC), Academy for Advanced Interdisciplinary Studies, Beijing National Laboratory for Molecular Sciences, College of Chemistry and Molecular Engineering, Peking University, Beijing, 100871 China; 3 Tsinghua-Berkeley Shenzhen Institute (TBSI), Tsinghua University, Shenzhen, 518055 China; 4 State Key Laboratory of Advanced Welding and Joining, Harbin Institute of Technology, Harbin, 150001 China; 5 Key Laboratory for the Physics and Chemistry of Nanodevices, Department of Electronics, Peking University, Beijing, 100871 China; 6 Electron Microscopy Laboratory, and International Center for Quantum Materials, School of Physics, Peking University, Beijing, 100871 China; 7 Collaborative Innovation Center of Quantum Matter, Beijing, 100871 China

## Abstract

Monolayer transition metal dichalcogenides (TMDs) have become essential two-dimensional materials for their perspectives in engineering next-generation electronics. For related applications, the controlled growth of large-area uniform monolayer TMDs is crucial, while it remains challenging. Herein, we report the direct synthesis of 6-inch uniform monolayer molybdenum disulfide on the solid soda-lime glass, through a designed face-to-face metal-precursor supply route in a facile chemical vapor deposition process. We find that the highly uniform monolayer film, with the composite domains possessing an edge length larger than 400 µm, can be achieved within a quite short time of 8 min. This highly efficient growth is proven to be facilitated by sodium catalysts that are homogenously distributed in glass, according to our experimental facts and density functional theory calculations. This work provides insights into the batch production of highly uniform TMD films on the functional glass substrate with the advantages of low cost, easily transferrable, and compatible with direct applications.

## Abstract (web summary)

Growth of large-area monolayer transition metal dichalcogenides is critical for their application but remains challenging. Here Yang et al. report rapid chemical vapor deposition of 6-inch monolayer molybdenum disulfide by sufficiently uniformly supplying the precursors and catalysts.

## Introduction

Recently, two-dimensional transition metal dichalcogenides (TMDs) have opened new perspectives for engineering next-generation electronics and optoelectronics, thanks to their unique physical and chemical properties different from their bulk counterparts^1–5^. In particular, direct band gap semiconductors, such as MoS_2_ and WS_2_, exhibit ultrahigh optical responsivity^6^, efficient valley polarization^7,8^ and strong light–matter coupling^9^, making them highly promising materials for constructing electrical/optical and energy-related devices. For such applications, the controllable synthesis of large-area uniform and large-domain monolayer TMDs is highly desired, while still challenging. This challenge is similarly encountered in graphene researches, and many efforts have been made in recent decades^10^.

To date, various techniques have been developed to synthesize TMDs, including physical vapor deposition^11^, metal organic chemical vapor deposition^12,13^, chemical vapor deposition (CVD)^14,15^, etc. Among these, the CVD has been recognized as the most promising route for directly synthesizing large-area uniform multi- or monolayer TMD materials^16–25^. Specifically, three strategies have been employed to deliver the metal precursors during the CVD process: the “pre-deposited” route, “point-to-face” supply, and “face-to-face” feeding methods. Taking the synthesis of MoS_2_ as an example, pre-deposition of Mo layers and MoO_2_ microcrystals followed by sulfurization processes were developed by Zhan et al.^17^ and Wang et al.^18^, respectively, for realizing the large-area growth of thin-layer MoS_2_ films. However, the TMDs usually evolved as polycrystalline layers due to limited migration/diffusion of metal precursors on macroscopic-scale surfaces. Subsequently, a point-to-face metal-precursor supply method was proposed to grow large-domain TMDs with controllable thicknesses. Lee et al. reported the successful synthesis of MoS_2_ atomic layers on SiO_2_/Si by mounting the substrate face-down over the MoO_3_ powder and sulfur precursors^20^. In addition, the substrate was also spin-coated with graphene-like molecules to promote the layered growth of MoS_2_. Without using seeds, Najmaei et al. demonstrated the synthesis of triangular domains and continuous MoS_2_ films by placing MoO_3_-nanoribbon-covered plate on the silicon substrate to reveal the nucleation and grain boundary formation mechanism^26^.

Notably, a point-to-face metal-source supply method has shown great potential for obtaining large-domain TMD monolayers using metal oxides (e.g, MoO_3_, MoO_2_) precursors placed upstream of (or below) the substrates. However, achieving large-scale uniform monolayer TMDs (over several centimeters) remains challenging, due to the variable release rates of the metal precursor and its inhomogeneous distribution along the gas-flow direction^27,28^. To guarantee a sufficient and uniform delivery of the metal precursor, a face-to-face deposition method was then proposed. Specifically, Yun et al. reported the direct synthesis of 2 × 3 cm^2^ uniform monolayer WS_2_ films by arranging (NH_4_)_6_H_2_W_12_O_40_·*x*(H_2_O) (AMT)-loaded Al_2_O_3_ plate above Au substrates^29^. However, the feeding rate of the precursors achieved through the two-step solution-processed assembly was still uncontrollable, and the method was too tedious to apply. Chen et al. reported the fast growth of millimeter-size monolayer MoSe_2_ crystals on molten glass^22^. A piece of Mo foil placed on SiO_2_/Si was used to hold the glass substrate. Very recently, the same group achieved the homoepitaxial growth of MoS_2_ patterns on monolayer MoS_2_ at a growth temperature of 1050 °C^23^. In the growth process, a piece of curved Mo foil was placed above the molten glass substrate serving as the Mo source. The ionic compounds in glass corroded the Mo foil thus helping with the volatilization of Mo, and resulting in a face-to-face metal-precursor feeding pathway.

Herein, we design a facile face-to-face metal-precursor supply strategy in the conventional CVD growth process, for synthesizing large-scale uniform, monolayer MoS_2_ by selecting a novel solid glass substrate and using Mo foil and sulfur as precursors. Soda-lime glass is chosen as the substrate as it is cost-effective and scalable, thus suitable for the batch production of monolayer MoS_2_ films. Meanwhile, the coating of MoS_2_ on glass endows it with novel optical and catalytic properties. Particularly, the large-scale uniformity, crystallinity, and growth efficiency of MoS_2_ on soda-lime glass substrate are carefully evaluated via detailed characterizations from atomic to centimeter scales. The ultrafast growth of MoS_2_ with the aid of trace amount of Na catalysts from the glass substrate is also discussed, according to intensive experimental efforts and density functional theory (DFT) calculations. In addition, by exploiting the hydrophilicity of the glass substrate, we also develop an etching-free transfer process for transferring inch-scale MoS_2_ films onto target substrates. This work, hereby, provides novel insights into the batch production and transfer of macroscopic uniform TMD films, which will propel their practical applications in various fields.

## Results

### Face-to-face metal-precursor feeding route

**Fig. 1:**
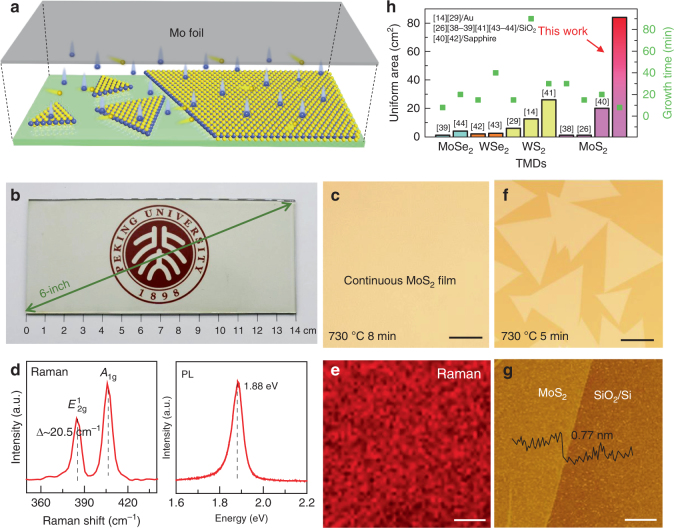
LPCVD growth of large-area uniform, monolayer MoS_2_ on soda-lime glass. **a** Schematic diagram of a face-to-face metal-precursor supply route. **b** Photograph of a 6-inch continuous MoS_2_ film on soda-lime glass synthesized for ~8 min. **c** Typical OM image of the MoS_2_ film, scale bar: 0.2 mm. **d** Raman (left panel) and PL (right panel) spectra of the as-grown MoS_2_, confirming its monolayer feature. **e** Raman mapping on the intensity of *A*_1g_ peak for the continuous MoS_2_ film, scale bar: 10 µm. **f** OM image of triangular MoS_2_ domains on glass grown for ~5 min with the other parameters kept identical. Scale bar: 0.1 mm. **g** AFM image of the edge of a MoS_2_ crystal (transferred onto SiO_2_/Si) and its corresponding height profile. Scale bar: 1 µm. **h** Comparison of the uniform area (bars) and growth time (green squares) of monolayer MoS_2_ on glass and other monolayer TMDs on various substrates reported in the literatures^14,26,29,38–44^

In order to obtain a highly uniform monolayer MoS_2_ film, we designed a unique face-to-face metal-precursor feeding route. As schematically illustrated in Fig. 1a, a Mo foil was placed above the soda-lime glass substrate in a parallel geometry with a gap of 10 mm. This configuration ensures a homogeneous supply of the Mo precursor through an onsite heating process with the aid of an O_2_ carrier (Supplementary Fig. 1). The concentration of the S precursor is usually oversaturated during the CVD growth process^30^. For the batch production purpose, commercial soda-lime glass (mainly composed of SiO_2_, Na_2_O, and CaO) was selected as the growth substrate considering the following factors: first, its low cost and scalability guarantees the cost-effective production of MoS_2_/glass hybrid materials; second, its hydrophilicity feature facilitates the transfer of MoS_2_ films to other substrates; third, the MoS_2_/glass hybrid itself may serve as a prototype material for directly fabricating optical and photoelectric devices due to its relatively high transparency (exceeds 91% at a wavelength *λ *= 550 mm). It is worthy of mention that the molten glass has been utilized as an ideal substrate for graphene growth in our previous efforts^31^. In another work, Ju et al. proposed the self-limited monolayer growth mechanism of MoS_2_ on molten glass for it is capable of trapping overflowing MoO_3_ precursors^32^. In contrast, the growth temperature in our system is set to 720 °C in order to preserve the original surface morphology of the glass substrate, thus providing higher potential for the batch production and the direct application of the hybrid material.

In the low-pressure (LP) CVD process, an adequate sulfur vapor was conveyed downstream by a flow of Ar (50 sccm) and O_2_ (6 sccm). The O_2_ carrier was introduced to oxidize the Mo foil for directly releasing chemically active MoO_3−*x*_ (*x* = 2, 3). This intermediate precursor presents relatively low sublimation temperature of about 500 °C, right below the melting point of the soda-lime glass. Notably, this temperature is much lower than that needed for the direct release of Mo atoms from Mo foils (~2600 °C). Generally, MoO_3−*x*_ is regarded as active species for the CVD synthesis of MoS_2_^19,33^. As shown in Fig. 1b, at relatively short growth time of 8 min, a 14 × 6 cm^2^ uniform, full-covered MoS_2_ film is synthesized on the glass substrate at 720 °C by a LPCVD strategy (see the Methods section). After this growth process, deformation of the glass substrate is negligible; however, its surface color changes from transparent to light brown, indicating the formation of MoS_2_.

According to the X-ray photoelectron spectroscopy (XPS) data, the Mo 3d_5/2_ (at 229.4 eV) and 3d_3/2_ (at 232.6 eV) peaks are in line with Mo^4+^ (Supplementary Fig. 2), and S 2p_3/2_ (at 162.2 eV) and 2p_1/2_ (at 163.4 eV) peaks are assigned to S^2−^. These results agree well with the standard XPS data for MoS_2_^18^, thus suggesting the formation of a MoS_2_ film. Moreover, the typical optical microscopy (OM) of the sample surface shows a highly uniform color contrast, indicating the homogenous thickness and in-plane continuity of the MoS_2_ film (Fig. 1c).

The layer thickness and crystal quality of the achieved MoS_2_ film were then determined by Raman spectroscopy and photoluminescence (PL) analyses. As shown in Fig. 1d, the randomly selected Raman spectra (collected from the 100 sampling points on the MoS_2_-covered glass) exhibit two characteristic peaks of MoS_2_, corresponding to the *E*^1^_2*g*_ (at 385.7 cm^−1^) and *A*_1g_ (at 406.2 cm^−1^) vibration modes, respectively. The specific frequency difference (∆) is 20.5 cm^−1^, highly indicative of the monolayer nature of the obtained film^34^. Moreover, the typical PL spectrum presents a sharp excitonic *A* peak at 1.88 eV (660 nm), its relatively high intensity and narrow full-width at half-maximum (54 meV) confirm the rather high crystallinity of our CVD-derived monolayer MoS_2_^35^. Moreover, Raman mapping on the intensity of *A*_1g_ mode manifests an extra uniform color contrast, further demonstrating its perfect thickness uniformity and good crystallinity over a large scale (Fig. 1e).

In order to investigate the intrinsic crystal quality, the growth time was deliberately reduced to 5 min. Large triangular domains are observed to be evenly dispersed on the surface by OM images, presenting an average edge length of ~200 µm (Fig. 1f and Supplementary Fig. 3). In this regard, the continuous film is proposed to arise from the merging of composite domains, either by extending the growth time or by increasing the precursors feeding rate. Notably, in a continuous monolayer film, the average domain size obtained in this work is much larger than previously reported ones synthesized on SiO_2_/Si^26,28^ or on Au foils^36^.

Interestingly, the domain size of the triangular MoS_2_ crystals is tunable by controlling the gap distance between the Mo foil precursor and the glass substrate. When the gap is decreased from 50 to 10 mm, the nucleation density reduces dramatically, and the average edge length of the resulting domains increases from 1 to 400 µm. This phenomenon is attributed to the gradual reduction in sulfur concentration over the gap region, which suppresses the nucleation density and increases the domain size accordingly. However, when the gap is further reduced to 2 mm, irregularly shaped MoO_*x*_S_2−*x*_ crystals appear on the sample surface due to an insufficient feeding of the S precursor, similar to the observation from the published reference^37^ (Supplementary Fig. 4). In addition, the MoS_2_ domains also increase in size with increasing concentration of O_2_ (from 1 to 6 sccm), due to the gradually increased oxidization of the Mo foil and sufficient supply of the metal precursor (Supplementary Fig. 5).

In order to accurately determine the thickness of the MoS_2_ film, a modified polymethyl methacrylate (PMMA)-assisted method was also developed to transfer the as-grown MoS_2_ film onto the SiO_2_/Si substrate, which will be discussed later. Figure 1g shows an atomic force microscopy (AFM) image of a MoS_2_ domain edge, and the corresponding height profile reveals a value of 0.77 nm, the same as that of the previously reported data for a monolayer thickness^20^. Notably, using the current face-to-face metal-precursor feeding route, the thickness uniform region is much larger than that of previously reported ones using the pre-deposited and point-to-face metal-precursor supply methods (Fig. 1e)^14,26,29,38–44^. For the batch production capability, the maximum sample size is only limited by the diameter of the tube furnace (3-inch diameter for the current experiment); larger sample size is attainable by increasing the size of the furnace.

**Fig. 2:**
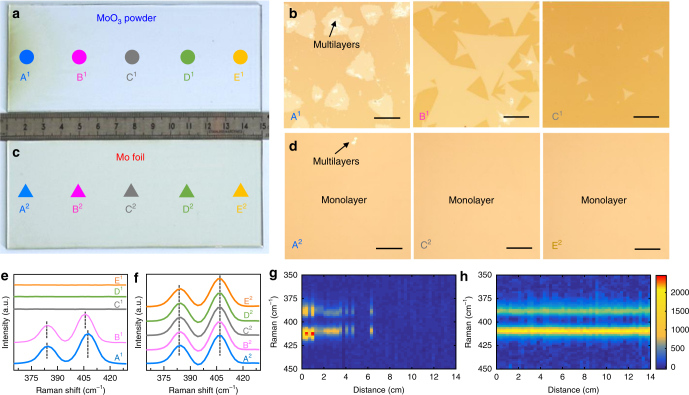
Comparison of MoS_2_ synthesized by a point-to-face and face-to-face metal-precursor supply routes. **a**, **c** Photographs of MoS_2_ growth on 6-inch soda-lime glass using **a** MoO_3_ powder and **c** Mo foil as precursors. The gas-flow direction was from left to right in both cases. **b**, **d** Corresponding OM images of MoS_2_ synthesized using **b** MoO_3_ powder and **d** Mo foil as precursors, at the points labeled A^1^, B^1^, and C^1^ in **a** and A^2^, C^2^, and E^2^ in **c** (with the different locations marked by colored letters; scale bars: 100 µm). **e**, **f** Corresponding Raman spectra of MoS_2_ synthesized with the MoO_3_ powder and the Mo foil precursors, respectively, at the points labeled A–E in **a** and **c**. **g**, **h** Color-coded images of the typical Raman modes for the samples shown in **a** and **c**, respectively, collected from 70 positions (with an interval of 2 mm along the horizontal direction)

To highlight the excellent thickness uniformity of the 6-inch monolayer MoS_2_ film on glass synthesized through a face-to-face metal-precursor feeding method, a point-to-face feeding route with the MoO_3_ powder precursor was also utilized to grow MoS_2_ on glass for comparison. Digital photographs of two pieces of 6-inch MoS_2_/glass samples, synthesized with the MoO_3_ powder placed upstream and the Mo foil placed on top, are displayed in Fig. 2a and c, respectively. Notably, the different thickness uniformity of the two-type samples is visible even to the naked eye. A “point-to-face” route-derived MoS_2_/glass shows gradually fading brown color along the gas-flow direction (*d* = 1–8 cm, where *d* denotes the distance from the left edge of the sample shown in the photograph), and the glass surface is almost colorless at the downstream location *d* = 8–14 cm. More specifically, the corresponding OM images of the selected points A^1^, B^1^, and C^1^ (marked in Fig. 2a with circles, corresponding to *d* = 1, 4, and 7 cm, respectively), present the characteristic morphologies of multilayer domains, larger monolayer domains (edge length 300 µm), and smaller monolayer domains (edge length 40 µm), respectively.

On the other hand, nearly homogenous contrast (uniform light-brown color) is noticeable over the entire face-to-face route-derived sample (Fig. 2c). Corresponding OM images of the points marked with A^2^, C^2^, and (shown by triangles in Fig. 2c) for *d* = 1, 4, and 13 cm, respectively, reveal a highly uniform color contrast over the entire surface, reconfirming the centimeter-scale uniformity of the obtained monolayer MoS_2_ films. To address the remarkably different thickness uniformity of the two-type samples, Raman spectra were also recorded at the five points (A to E) indicated in Fig. 2a, c. For a point-to-face precursor-feeding method, ∆ of the two characteristic peaks (*E*^1^_2*g*_ and A_1g_) decreases from ~22.6 to ~20.7 cm^−1^ along the upstream points (positions A^1^ and B^1^ in Fig. 2a), consistent with the thickness change from multilayer to monolayer (also shown in the OM images). In addition, only a faint Raman signal is visible at the downstream points (positions C^1^, D^1^, and E^1^ in Fig. 2a) due to the few nucleation sites.

However, the Raman spectra for a face-to-face precursor-feeding derived sample are nearly identical for various locations along the sample, with the characteristic ∆ value of 20.5 cm^−1^, highly indicative of its superior thickness uniformity (Fig. 2f). Moreover, Raman data from 70 typical locations (with an interval of 2 mm) were also collected in Fig. 2g and h for the two-type samples. Clearly, a “face-to-face” precursor-feeding derived sample exhibits excellent thickness uniformity in peak intensity and frequency difference (∆ of 20.6 ± 0.3 cm^−1^).

The disparate film thickness uniformities of the two-type MoS_2_/glass samples (synthesized using a face-to-face and point-to-face metal-precursor feeding routes) can be explained by the different concentration gradients of the Mo species over the substrate surfaces. In the latter case, the MoO_3_ powder is partially reduced by the sulfur vapor to form volatile MoO_3−*x*_ species, which are subsequently transported downstream by the gas flow and react with sulfur^19^. Apparently, the concentration of the MoO_3−*x*_ species gradually decreases along the gas-flow direction, leading to smaller and sparser MoS_2_ crystals at the downstream positions. However, in our designed growth strategy, wherein the Mo foil is placed face-to-face to the glass substrate, the MoO_3–*x*_ species arising from the oxidized Mo foil can be uniformly released into the gap between Mo foil and glass, resulting in homogenous nucleation and growth over the entire glass surface. Accordingly, a large-area uniform monolayer MoS_2_ film is unexceptionally obtained.

### Growth mechanism of monolayer MoS_2_ on soda-lime glass

**Fig. 3:**
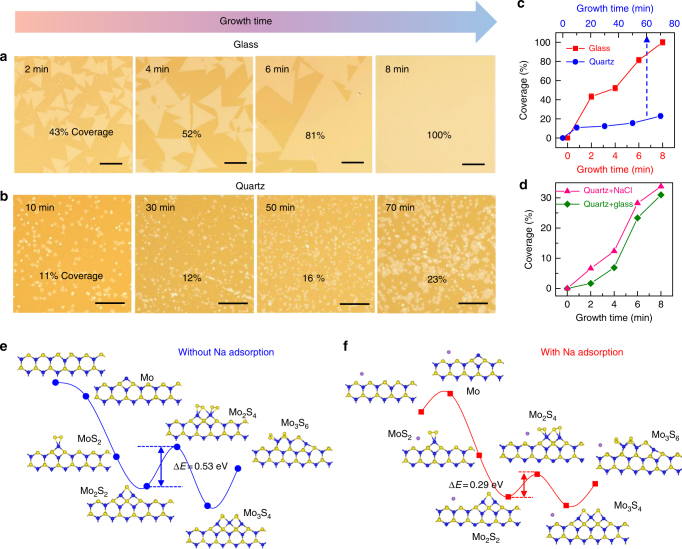
The role of Na from soda-lime glass in promoting the growth rate of monolayer MoS_2_. Growth-time**-**dependent OM images of MoS_2_ synthesized on **a** soda-lime glass with growth time from 2 to 8 min (scale bars: 100 µm) and **b** quartz glass from 10 to 70 min (scale bars: 50 µm). Other than the substrate type, all experimental parameters were identical for the samples shown in **a** and **b**. **c** MoS_2_ coverage as a function of growth time on soda-lime glass (red) and quartz (blue). **d** MoS_2_ coverage as a function of growth time on quartz substrates either placed downstream from the glass (green) or spin-coated with NaCl (pink) prior to growth. **e**, **f** DFT-calculated energy diagrams for MoS_2_ growth along the S-terminated edges, **e** without and **f** with Na adsorption. The blue, yellow, and purple spheres represent molybdenum, sulfur, and sodium atoms, respectively

In our experiments, the continuous 6-inch monolayer MoS_2_ film is usually synthesized on glass at a quite short time of 8 min, in line with an edge growth rate of around 1.2 µm s^−1^. Notably, this growth rate is much faster than that on common insulating substrates, such as SiO_2_/Si (15 min; 0.4 µm s^−1^)^26^ and sapphire (30 min; 0.2 µm s^−1^)^24^. The glass substrate, thereby, plays a crucial role in the fast growth of monolayer MoS_2_ films. To understand this, the MoS_2_ growth both on soda-lime glass (mainly composed of SiO_2_, Na_2_O, and CaO) and quartz (mainly composed of SiO_2_) were compared in detail. Typical OM images of MoS_2_ grown for different time durations on soda-lime glass (2–8 min) and on quartz (10–70 min) are presented in Fig. 3a and b, respectively. This growth time difference should justify their different growth rates in general. As for the surface morphology, well-defined triangular flakes with an edge length of hundreds of microns can be noticed on glass, and the coverage is precisely tunable from 43 to 100% with increasing growth time from 2 to 8 min (with an interval of 2 min) (Fig. 3a, from left to right). In contrast, when using the same growth parameter, there is much poorer control of the morphology of MoS_2_ on quartz with the growth time varying from 10 to 70 min (with an interval of 20 min) (Fig. 3b, from left to right). The sample is characterized with much smaller domain sizes (edge lengths 5–10 μm), high density of nucleation sites, and nonuniform thicknesses. Moreover, according to statistical results, the coverage of MoS_2_/glass increases monotonically with growth time, and the growth rate is approximately 38 times higher than that on quartz (Fig. 3c; as calculated from the coverage expansion per minute). Intriguingly, by placing a piece of soda-lime glass on the upstream area of the quartz substrate, the domain size and the growth rate of MoS_2_ on quartz are significantly increased (Supplementary Fig. 6). Considering the different chemical compositions of soda-lime glass and quartz, the metallic elements in glass, e.g., Na and Ca, should take effect in the fast growth of MoS_2_.

To confirm this, a quartz substrate was spin-coated with a solution of CaCl_2_ (0.01 g ml^−1^) or NaCl (0.01 g ml^−1^) before depositing MoS_2_. The derived MoS_2_ layer on the CaCl_2_-coated quartz exhibits nonuniform thicknesses and irregular domain shapes (Supplementary Fig. 7), indicating that the effect of Ca is negligible for accelerating the growth of MoS_2_. However, for the quartz substrate coated with NaCl, both the domain size and surface coverage of MoS_2_ are significantly enhanced (Fig. 3d and Supplementary Fig. 6). Statistically, the average growth rate of MoS_2_ on quartz with the assistance of upstream glass or spin-coated with NaCl is approximately 13 times faster than that on pure quartz (Fig. 3d).

In order to exclude the contribution of Cl, quartz spin-coated with NaOH solution (0.01 g ml^−1^) was also employed as a substrate for MoS_2_ growth. The result is quite similar to that for NaCl-coated substrate (Supplementary Fig. 8). Based on these experiments, the Na element in the soda-lime glass is considered to serve as a very effective promotor in the fast growth of MoS_2_. As an additional proof, Na is also detected in the oxidized Mo foil after MoS_2_ growth, as evidenced by XPS spectra (Supplementary Fig. 9). Thus, Na is expected to be widely distributed in the confined space between Mo foil and glass during the CVD growth process. However, after being transferred onto SiO_2_/Si, no Na signal appears for the monolayer MoS_2_ film, confirming that Na only serves as an intermediate catalyst for the rapid growth of MoS_2_ (Supplementary Fig. 9).

**Fig. 4:**
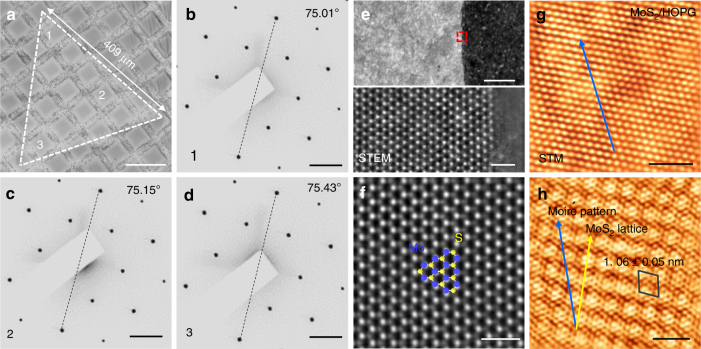
Atomic structure of transferred monolayer MoS_2_. **a** OM image of a transferred MoS_2_ domain on a carbon TEM grid, scale bars: 100 µm. **b**–**d** SAED patterns from the regions numbered 1–3 in **a**. The dashed lines indicate the rotation angles (75.01°, 75.15°, and 75.43°) with respect to the horizontal line (scale bar: 3 nm^−1^). **e** STEM (upper) image of the MoS_2_ domain edge and the enlarged view (lower) of the marked rectangular region. Scale bars: 20 and 1 nm, respectively. **f** Atomically resolved image representing the defect-free hexagonal structure of MoS_2_. The bright spots are Mo atoms and the gray spots correspond to two-stacked S atoms. Scale bars: 1 nm. **g** Representative Moiré-scale STM image of MoS_2_ transferred on HOPG (*V*_T_ = −0.21 V, *I*_T_ = 5.19 nA, scale bar: 5 nm) and **h** corresponding atomic-resolution STM image (*V*_T_ = −0.33 V, *I*_T_ = 5.19 nA, scale bar: 2 nm). The arrows indicate the directions of the MoS_2_ lattice (yellow) and the Moiré pattern (blue). The unit cell for the Moiré pattern is outlined with a rhombus (period of 1.06 ± 0.05 nm)

DFT calculations were then performed to provide an in-depth understanding of the growth mechanism. The energy diagrams for MoS_2_ growth along the S-terminated edges were calculated with and without Na adsorption (for the first six reaction steps), as displayed in Fig. 3e and f, respectively. With the incorporation of Na, the highest energy barrier (that of the step from Mo_2_S_2_ to Mo_2_S_4_) reduces from 0.53 to 0.29 eV for MoS_2_ growth. Assuming that the rates of atom attachment (S_2_ and Mo) are the same for the two cases, the ratio of growth rate with Na to that without Na is estimated as exp[(0.53–0.29)*k*^−1^*T*^−1^] ≈ 17 at *T* = 1000 K. This result is in good agreement with the experimental data shown in Fig. 3c. Meanwhile, for the growth of MoS_2_ along the Mo-terminated edge (Supplementary Fig. 10), the energy barriers are calculated to be 1.29 and 2.04 eV for those with and without Na assistance, respectively. Much faster growth of the S-terminated edge indicates the preferential evolution of Mo-terminated edges according to the published reference^27^, which corroborates our experimental results (as will be discussed in Fig. 4e).

### Atomic structure characterizations of the crystal quality

Transmission electron microscopy (TEM), scanning transmission electron microscopy (STEM), and scanning tunneling microscopy (STM) were then utilized to evaluate the crystal quality of the CVD-derived monolayer MoS_2_. Figure 4a shows an OM image of a typical triangular MoS_2_ domain with an edge length of 409 µm. Selected area electron diffraction (SAED) patterns regarding the numbered regions (1, 2, and 3 in Fig. 4a) present nearly identical lattice orientations (deviation smaller than ± 0.5°), suggesting the single-crystal nature of the MoS_2_ triangular domain (Fig. 4b–d). Moreover, the atomically resolved image from the domain edge presents a Mo-terminated zigzag edge type (Fig. 4e), which agrees well with the previously published result^28^. Specifically, the lattice inside the domain presents a well-organized honeycomb lattice with an interatomic distance of approximately 0.32 nm (Fig. 4f), which is identical to that previously documented for MoS_2_^45^. And the nearly defect-free structure confirms the rather high crystal quality of the CVD-derived MoS_2_ on glass.

In order to present the relatively high stability and perfect crystal quality, the monolayer MoS_2_ flake was transferred onto a highly oriented pyrolytic graphite (HOPG) substrate for STM characterizations. Large-area uniform, hexagonal Moiré patterns are universally observed to show a period of 1.06 ± 0.05 nm (Fig. 4g, as marked by a rhombus in Fig. 4h), which arises from the lattice mismatch between MoS_2_ (*a*_1_ = 0.312 nm) and graphite (*a*_2_ = 0.246 nm). Briefly, STEM and STM observations provide consistent proof of the relatively high crystal quality of CVD-derived monolayer MoS_2_, as well as its perfect stability during the harsh sample transfer process.

### Green transfer and electronic property characterizations

To fulfill the applications of large-area uniform monolayer MoS_2_, a convenient transfer process is highly desirable. To date, two-predominant strategies using PMMA as the supporting layer have been demonstrated, i.e., electrochemical bubbling and wet chemical etching methods. The former is mainly used to transfer monolayer TMDs on conductive substrates, such as Au foils, by virtue of the weak interfacial interaction^14,29^. In the latter case, structural damage and performance degradation usually occur due to the use of acidic and alkaline solutions^44^. To mitigate this issue, facile/rapid, environmentally friendly transfer routes need to be developed for specific growth systems. Recently, Gurarslan et al.^46^ and Xu et al.^47^ have developed similar methods to transfer MoS_2_ and WS_2_ from sapphire, based on the hydrophilic behavior of the sapphire substrate and the accompanying capillary force. For the former work, the polymer/MoS_2_ stack needed to be picked up from the water droplet and then transferred onto target substrates, which can hardly ensure the large-scale continuity of the large-size samples. For the latter work, a NaOH solution pre-etching process was highly necessary to open a gap between polymer and sapphire, which inevitably caused damage to both sample and substrate.

**Fig. 5:**
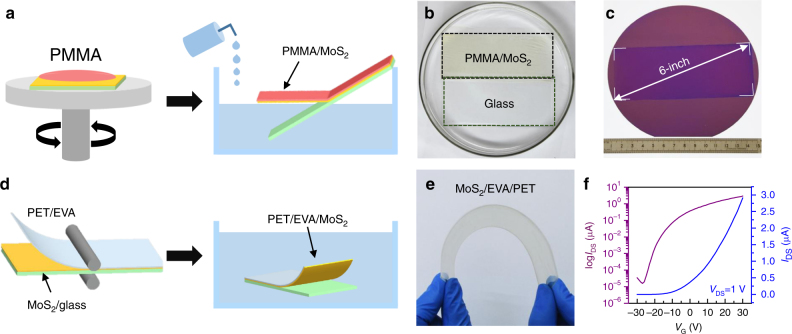
Green transfer process and device performance of monolayer MoS_2_. **a** Schematic diagram of the PMMA-assisted etching-free transfer process onto rigid substrates. **b** Photograph of the delamination process for removing the PMMA/MoS_2_ film from the glass substrate. **c** Photograph of the 6-inch MoS_2_ film transferred onto a SiO_2_/Si substrate. **d** Schematic diagram of the roll-to-roll transfer route onto flexible substrates. **e** Photograph of a typical 6-inch uniform, flexible, monolayer MoS_2_/EVA/PET stack. **f** *I*_DS_–*V*_G_ curves of a typical MoS_2_ FET device at *V*_DS_ = 1 V

In this work, by virtue of the hydrophilicity of the glass substrate, an etching-free, easy processing, and scalable transfer strategy was developed for the high-quality transfer of large-area uniform samples (Supplementary Fig. 11). As illustrated in Figs. 5a and d, both PMMA-assisted and the roll-to-roll transfer strategies were designed to transfer MoS_2_ from glass to SiO_2_/Si and ethylene vinyl acetate/polyethylene terephthalate (EVA/PET) substrates, respectively. For the transfer to rigid substrates, a modified PMMA-assisted transfer process involving three essential steps was developed: first, spin-coating PMMA on MoS_2_/glass; then making cracks along the edge of PMMA/MoS_2_ with a knife, inducing natural penetration of water into the interface between MoS_2_/PMMA and substrate due to their different surface energies (Supplementary Fig. 11); finally, inclining the PMMA/MoS_2_/glass inside a container and injecting water at an optimized rate of 10 ml s^−1^ to minimize the interface stress. In this way, the 6-inch PMMA/MoS_2_ film can be easily delaminated from the glass substrate, mainly relying on the interfacial capillary force (Fig. 5b). Finally, the PMMA/MoS_2_ film was fished by the target substrate and then immersed in acetone to remove PMMA (Fig. 5c). Interestingly, the entire peel-off process can be completed within 60 s for a 6-inch MoS_2_ film. Directly injecting water rather than gradually feeding the sample into water is much easier to handle and is more suitable for transferring large-area samples.

Compared to the PMMA-assisted transfer route, the developed roll-to-roll method was more convenient as follows: adhesion of EVA/PET plastic onto MoS_2_/glass via a portable hot lamination process, which is also used to transfer graphene^48^; immersion of the EVA/PET/MoS_2_/glass into the deionized water, thus induces automatically penetration of water into the interface between PET/EVA/MoS_2_ and the glass substrate, followed with effective delamination within 5 min for a 6-inch sample (Fig. 5e).

Notably, both the PMMA-assisted and roll-to-roll transfer pathways are free of acid or alkaline etching, thus avoiding unnecessary contamination and irreversible damage, either to the MoS_2_ film or to the glass substrate. Accordingly, these carefully designed transfer routes can preserve the original high quality of the CVD-derived monolayer MoS_2_, and they are more environmentally friendly, efficient, and easier to operate than the commonly used wet chemical etching route. This intact transfer route also guarantees the recyclable use of the glass substrate, as presented in Supplementary Fig. 12 by Raman spectra and OM images. Even so, the soda-lime glass substrate should be used for less than three times considering the gradually decreased Na content on the glass surface.

To evaluate the electrical performance of MoS_2_ samples, back-gated field-effect transistors (FETs) were fabricated on 300 nm SiO_2_/Si substrates based on transferred MoS_2_ monolayers with 10 nm Ti/50 nm Au as source and drain electrodes (Supplementary Fig. 13). The transfer characteristic curve of a typical FET device is presented in Fig. 5f, with a channel length of 1 µm and a channel width of 3 µm, which shows a typical n-type behavior. In addition, the transport characteristics of 46 randomly selected MoS_2_ FET devices are also analyzed (Supplementary Fig. 13). The achieved mobility and on/off ratio of these devices falls in a narrow range of 6.3 to 11.4 cm^2^ V^−1^ s^−1^ and 10^5^ to10^6^, respectively, suggesting the relative high-quality uniformity of our large-scale MoS_2_ samples. Both parameters are comparable to those of back-gated FETs fabricated with CVD-grown MoS_2_ flakes^20,24,26,28,32,49–52^ (Supplementary Table 1). Notably, the carrier mobility can be further improved by optimizing the interface contact by pre-treatment^24^ or changing source/drain electrodes^53,54^.

## Discussion

In summary, by exploiting a face-to-face metal-precursor supply method, we have successfully synthesized a 6-inch uniform monolayer MoS_2_ film with the domain size larger than 400 µm on solid soda-lime glass. Particularly, the sample size is even scalable by increasing the size of the growth chamber, considering the rather homogenous precursor feeding route designed in this work. Intriguingly, the uniformly distributed Na in soda-lime glass is confirmed to serve as perfect catalysts for the rapid and large-scale uniform growth of monolayer MoS_2_, according to both experimental facts and DFT calculations. By exploiting the hydrophilicity feature of glass, we have also developed an etching-free method to transfer large-area MoS_2_ films onto targeted substrates. We believe that this work should pave the way for the cost-effective batch production, environment-friendly transfer, as well as versatile applications in both fundamental and industrial aspects.

## Methods

### Face-to-face metal-precursor feeding assisted synthesis of macroscopic uniform monolayer MoS_2_ on soda-lime glass

Before synthesis, the commercial soda-lime glass (14 × 6 cm^2^; 2 mm thick) was cleaned in deionized water to remove surface impurities. A rectangular piece of Mo foil (99.95%; 0.025 mm thick) was folded into a bridge and placed on top of the soda-lime glass, as shown in Fig. 1a. The Mo foil and glass were placed in a graphite boat and loaded into a 3-inch CVD chamber. Another quartz boat containing sulfur powder (99.5%) was placed upstream, 40 cm away from the substrate. Before heating, the furnace system was purged with 80 sccm Ar for 10 min. Then, Ar (50 sccm) and O_2_ (6 sccm) gas flows were introduced into the system to create a stable growth atmosphere. The sulfur zone and the substrate zone were heated to 100 and 720 °C within 35 min, respectively. The growth was performed for 8 min to achieve the 6-inch uniform monolayer MoS_2_ film, and the furnace was then cooled to room temperature automatically.

### PMMA-assisted transfer of 6-inch monolayer MoS_2_ film

Firstly, the as-grown MoS_2_/glass was spin-coated with PMMA (950 K, Allresist, AR-P 679.04) at a speed of 80 rpm for 1 min followed by curing at 80 °C for 10 min. Then, the edge of PMMA film was scored with a knife to provide a path for the water penetration. Next, the PMMA/MoS_2_/glass was inclined against the edge of a petri dish, and water was injected with an optimized rate of 10 ml s^−1^, as shown in Fig. 5a. In this way, the PMMA/MoS_2_ stack was spontaneously delaminated from glass, and it was then collected by a target substrate. Finally, the PMMA coating was removed using acetone and dried under the flowing N_2_ gas. For TEM characterizations, the PMMA layer was dissolved with acetone droplets and dried naturally.

### Roll-to-roll transfer of monolayer MoS_2_ film on PET

The 6-inch uniform MoS_2_ film on soda-lime glass was hot laminated at 180 °C with EVA (50 µm thick) pre-coated with PET (75 µm thick) using a lamination machine to form a glass/MoS_2_/EVA/PET stack. Then, the glass/MoS_2_/EVA/PET film was soaked in deionized water for ~5 min. After delamination, the MoS_2_/EVA/PET stack was dried by flowing N_2_.

### DFT calculations

All calculations were performed adopting the Perdew–Burke–Ernzerhof^55^ parametrization for the generalized gradient approximation of the exchange-correlation effect embedded in the Vienna Ab initio Simulation Package^56,57^. The ion–electron interaction was described by the projector-augmented wave potentials^58,59^. Following the kink-flow scheme using a nanoreactor model for graphene^60^, S_2_ and Mo were introduced stepwise to the S- or Mo-terminated growth front. The growth fronts were modeled by zigzag nanoribbons of nine lattice units along the periodic direction. The energies of these nanoribbons with and without Na adsorption were taken as references for constructing the energy diagrams. To simplify the discussion, the chemical potential of S was obtained for the most stable sulfur molecule S_2_ at the growth temperature of 1000 K and partial pressure of 10 kPa, considering their transitional, rotational, and vibrational contributions^61^. Under such assumptions, the chemical potential of S_2_ was calculated to be about −10 eV, while those of the bulk material were used for Mo and Na. It should be noted that the choices of chemical potentials did not influence the relative energies for the two cases with and without Na adsorption. The adsorption energies of Na on Mo- and S-terminated edges were calculated as −0.82 and −2.09 eV, indicating their favorable incorporation into the growth fronts.

### Characterization of MoS_2_ films

OM (Nikon ECLIPSE, LV100ND), contact angle measuring system (Dataphysics, OCA20), Raman spectroscopy (HORIBA, LabRAM HR-800, with an excitation wavelength of 514 nm), AFM (Bruker, Dimension Icon), XPS (Kratos Analytical AXIS-Ultra with monochromatic Al Kα X-ray), TEM (JEOL JEM-2100F), STEM (Titan Cubed Themis G2), and ultra high vacuum low temperature (UHV LT) STM were used to characterize the optical and structural properties of the MoS_2_ sample.

### Data availability

The data reported by this article are available from the corresponding author upon reasonable request.

## Electronic supplementary material

Supplementary Information

Peer Review File
