# Supplementary material for: Batch production of 6-inch uniform monolayer molybdenum disulfide catalyzed by sodium in glass
Source: Nat Commun. 2018 Mar 7;9:979. doi: 10.1038/s41467-018-03388-5 (PMC5841402; doi:10.1038/s41467-018-03388-5)
Supplement: Supplementary file 1 — Supplementary Information [file 41467_2018_3388_MOESM1_ESM.pdf]

Batch production of 6-inch uniform monolayer molybdenum sulfide catalyzed by sodium in glass

Pengfei Yang[1,2], Xiaolong Zou[3], Zhepeng Zhang[1,2], Min Hong[1,2], Jianping Shi[1,2], Shulin Chen[2,4], Jiapei Shu[5], Liyun Zhao[1], Shaolong Jiang[1,2], Xiebo Zhou[1,2], Yahuan Huan[1,2], Chunyu Xie[1,2], Peng Gao[2,6,7], Qing Chen[5], Qing Zhang[1], Zhongfan Liu[2], Yanfeng Zhang[1,2]*

Supporting information

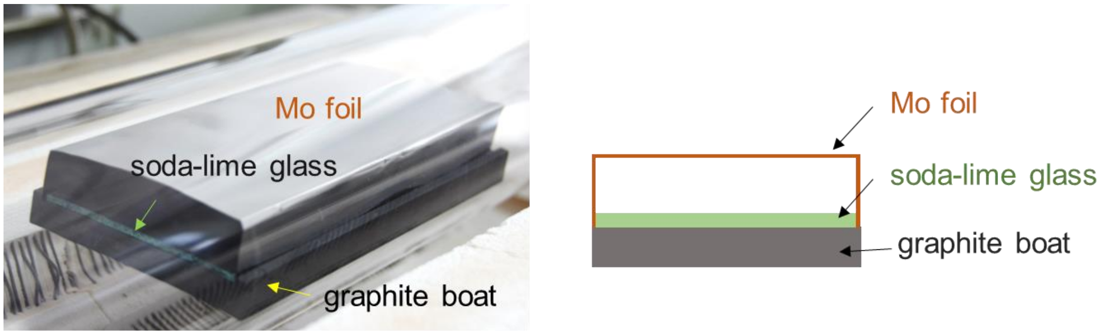

Supplementary Figure 1. Photograph of the face-to-face metal-precursor supply strategy for the growth of MoS_2 in a facile chemical vapor deposition (CVD) route. The graphite boat has a good wettability to glass. Hereby, the surface flatness of the glass substrate can be maintained even after higher temperature growth than that of the melting point of soda-lime glass.

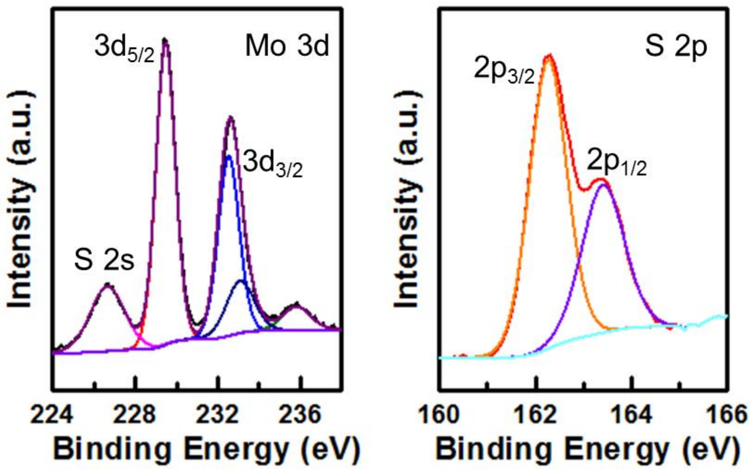

Supplementary Figure 2. XPS spectra of as-grown MoS_2 on glass. The characteristic peaks at 229.4 eV and 232.6 eV correspond to Mo $3d_{5/2}$ and $3d_{3/2}$, and the peaks at 162.2 eV and 163.4 eV are in line with $2p_{3/2}$ and $2p_{1/2}$, respectively. Other two characteristic peaks centered at 233.1 and 235.7 eV are assigned to the binding energies of Mo $3d_{5/2}$ and $3d_{3/2}$ of MoO_4^{2-}, considering the trapping effect of glass to the Mo precursors according to the published reference[1].

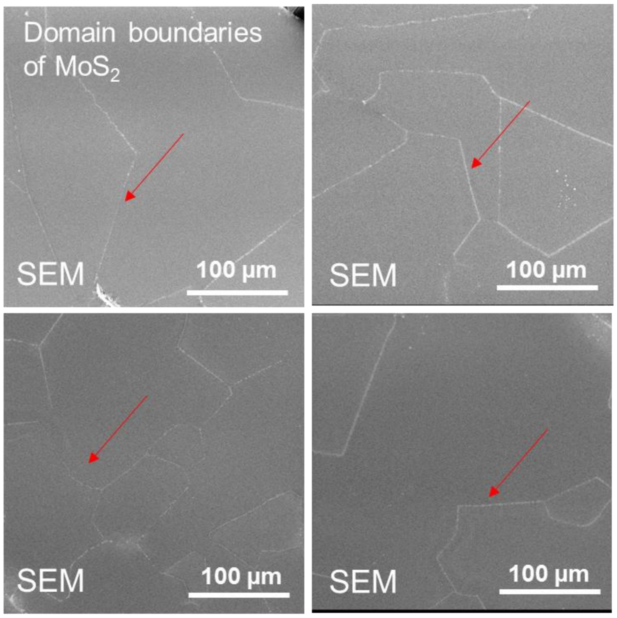

Supplementary Figure 3. Typical SEM images of the as-grown continuous monolayer MoS_2 film on glass after a mild oxidation process (80°C heating under moisture-rich ambient conditions; humidity 60% for 10 min)[2]. The domain boundaries can be easily recognized by their brighter contrasts with regard to that of the MoS_2 flakes.

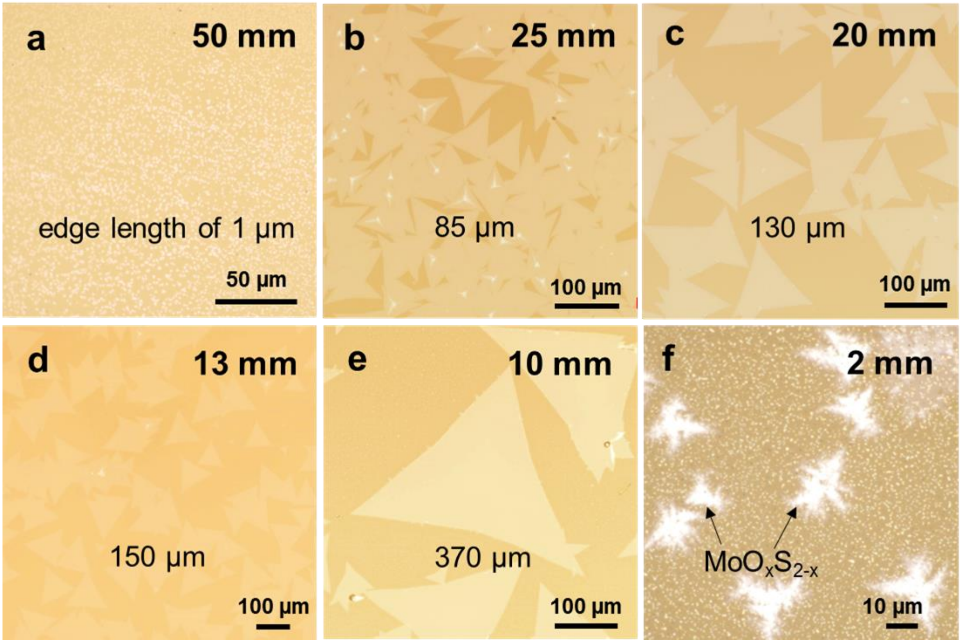

Supplementary Figure 4. The effect of the distance ($D_{pre\text{-}sub}$) between Mo foil precursor and glass substrate on the growth of monolayer MoS_2 on glass. **(a–f)** Optical images for MoS_2 growth with different $D_{pre\text{-}sub}$ of (a) 50 mm, (b) 25 mm, (c) 20 mm, (d) 13 mm, (e) 10 mm, and (f) 2 mm. All the other experimental conditions (T= 720 ° C， t = 6 min) are the same. Briefly, a suitable $D_{pre\text{-}sub}$ value is essential for the growth of large domain monolayer MoS_2 on glass.

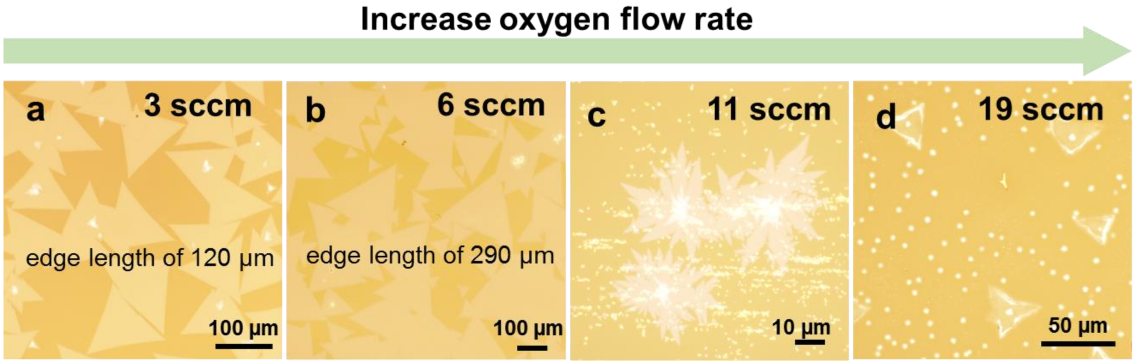

Supplementary Figure 5. The influence of O_2 concentration on the growth of MoS_2 on glass. **(a–d)** Optical images of the MoS_2 flakes synthesized with different O_2 concentrations: (a) 3 sccm, (b) 6 sccm, (c) 11 sccm, and (d) 19 sccm, respectively, at T= 720 °C, t= 8 min, $D_{pre\text{-}sub}$ = 10 mm. It can be found that, the domain size of MoS_2 can be improved, which is realized by lowering the O_2 concentration or by inducing a mild oxidization of the Mo foil, affording a suitable feeding of the volatile MoO_{3-x} species and a relative low nucleation density. However, when excess O_2 is introduced, the etching effect to the evolved MoS_2 flakes is more obvious, leading to small domain size or the formation of Mo oxides, the same as the data from the published reference[3].

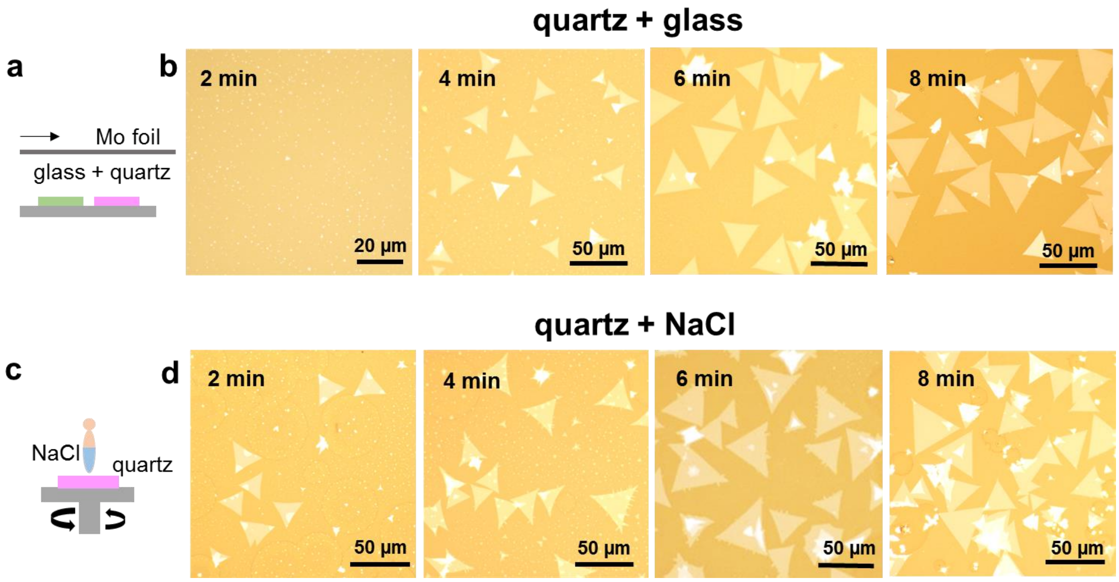

Supplementary Figure 6. Two alternative strategies for improving the growth rate of MoS_2 on quartz with the introduction of trace amounts of Na catalysts. **(a)** Schematic of the first design by placing quartz at the downstream location of glass. The distance between quartz and glass was 1 cm. **(b)** Corresponding OM images of MoS_2 with growth time of 2 to 8 min by the design in (a). **(c)** Schematic of the second design by spin-coating quartz with NaCl solution (0.01g ml^{-1}) and then air-drying prior to CVD growth. **(d)** Corresponding OM images of MoS_2 growth for 2 to 8 min on quartz (coated with NaCl particles). It is obvious to see that, the surface coverage and edge length of MoS_2 flakes grown on quartz through these two ways are similar with each other. Multilayer MoS_2 flakes primarily nucleate around the Na-based particles arising from the upstream glass or pre-coated NaCl, which distribute non-uniformly on the quartz surfaces.

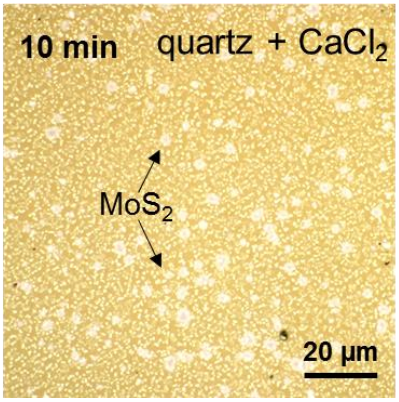

Supplementary Figure 7. OM image of the MoS_2 crystals synthesized on quartz spin-coated with $CaCl_2$ solution (0.01g ml^{-1}) and then air-dried prior to CVD growth. It can be found that, the MoS_2 flakes present irregular shapes with an average diameter of about 3 µm, which is close to the case for MoS_2 growth on bare quartz at the same growth time.

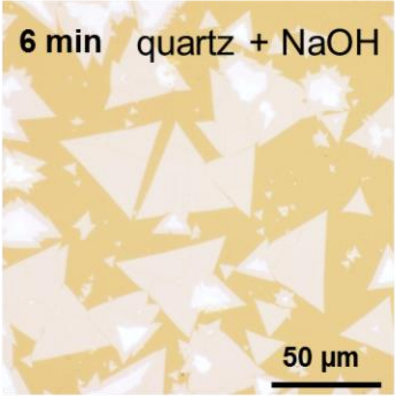

Supplementary Figure 8. OM image of the MoS_2 crystals synthesized on quartz (spin-coated with the NaOH solution (0.01 g ml^{-1}) and air-dried prior to growth). The edge lengths of MoS_2 reach 50 µm for 6 min growth, which is quite similar to the growth on quartz pre-coated with NaCl. The formation of multilayer MoS_2 is attributed to the existence of Na-based particles that inhomogeneously distributed on the glass substrate surface, which is catalytically active for the formation of MoS_2.

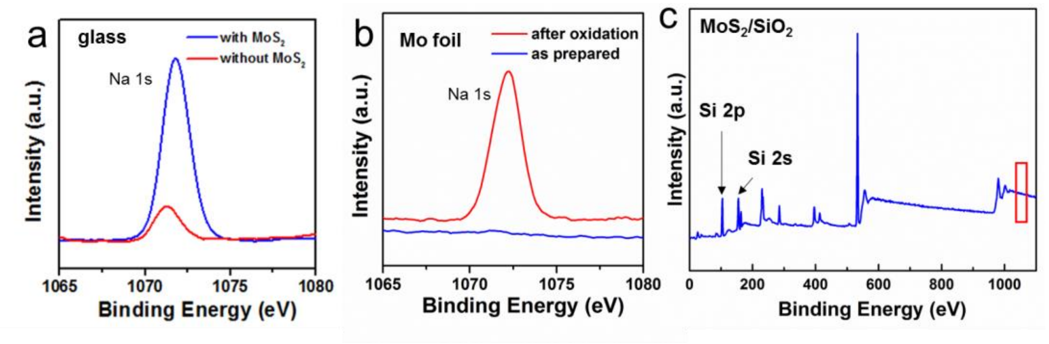

Supplementary Figure 9. XPS spectra of the Na 1s peak in **(a)** for the soda-lime glass surface before and after MoS_2 growth; **(b)** For Mo foil before and after MoS_2 growth; **(c)** MoS_2 transferred on the SiO_2/Si substrate. It is intriguing to find that, the Na 1s peak on glass is more intense after the growth of MoS_2 than that before growth, according to the XPS data shown in (a). This indicates that Na atoms accumulate on the surface of glass after growth. Moreover, an intense Na peak appears on the Mo foil after MoS_2 growth, which addresses that Na is widely distributed in the gap region, and adsorbed on the upper Mo foil surface after the CVD growth. After a normal sample transference process, the Na residual adsorbed on MoS_2 is washed away by the deionized water. Accordingly, Na serves as an intermediate catalyst for the growth of MoS_2 on glass.

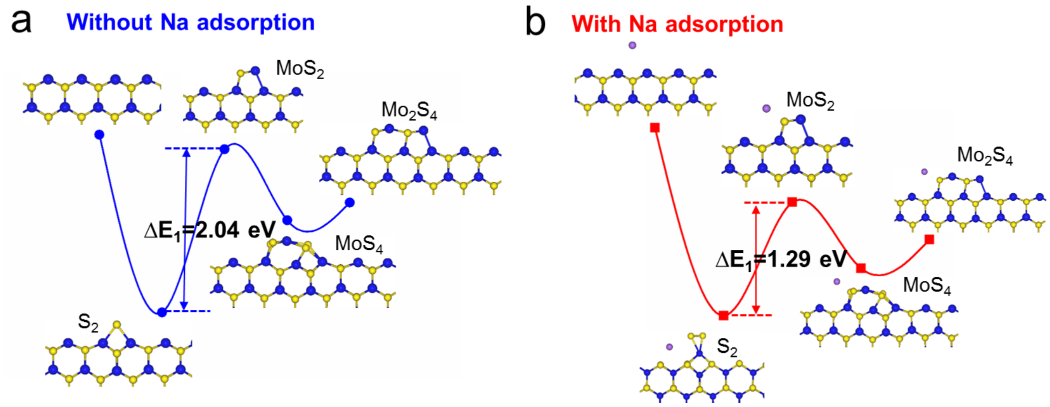

Supplementary Figure 10. DFT calculations of the energy diagrams for the growth of MoS_2 along the Mo-terminated edges: **(a)** without and **(b)** with the Na adsorption. Blue, yellow and purple spheres represent molybdenum, sulfur and sodium atoms, respectively. For MoS_2 growth along the Mo-terminated edge, the highest barrier (step from S_2 to MoS_2) reduces from 2.04 to 1.29 eV by combining the Na catalysts.

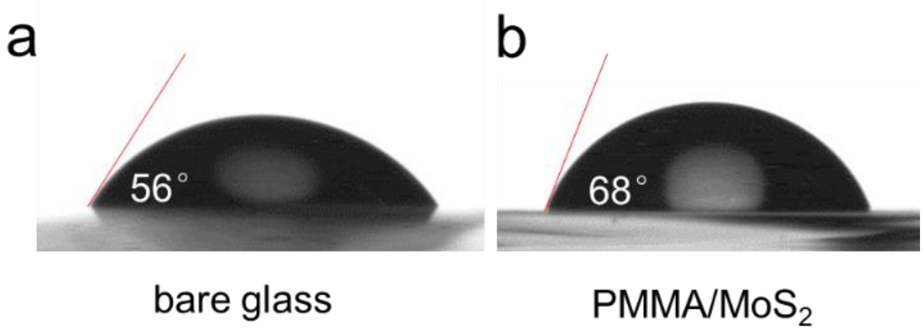

Supplementary Figure 11. Contact angle measurements of **(a)** bare glass and **(b)** PMMA/MoS_2. The glass substrate is hydrophilic, while the PMMA film is more hydrophobic. As a result, water tends to penetrate into the interface between MoS_2/PMMA and substrate due to their different surface energies.

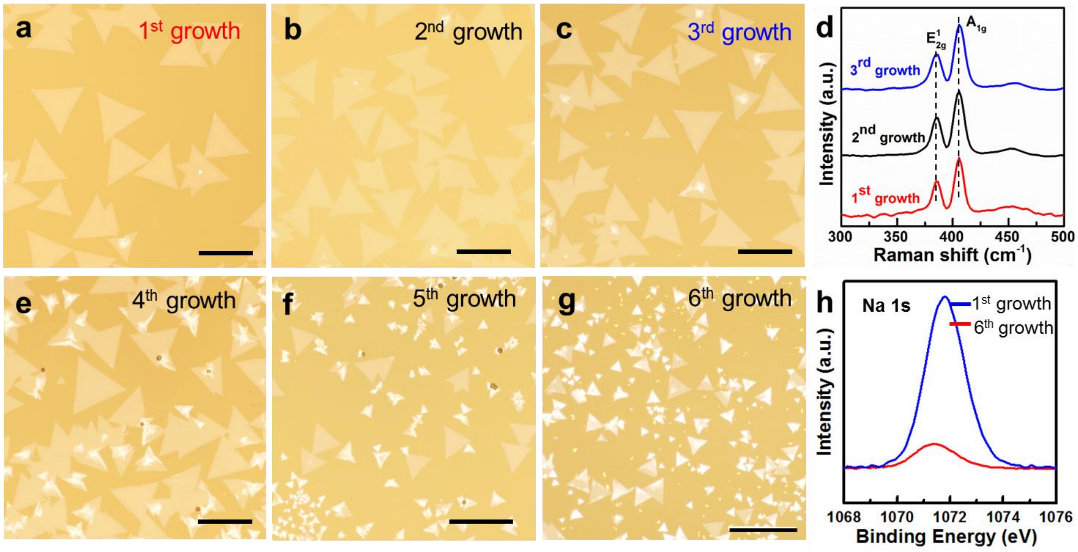

Supplementary Figure 12. (a-c) Optical images of the growth of MoS_2 on soda-lime glass used for three times. All the other experimental conditions (T= 720 °C, t = 3 min) are the same. Scale bars: 100 µm. **(d)** Raman spectra of MoS_2 synthesized on the glass used for three times. **(e-g)** Optical images of the growth of MoS_2 on soda-lime glass used for four to six times with the same growth condition. Scale bars: 100 µm. **(h)** XPS spectra of the Na 1s peak for the soda-lime glass after MoS_2 growth for 1st and 6th times, respectively. These results indicate that, the repeatable use of the glass substrate is probable for three times in the current growth system. However, when the glass substrate is used for four to six times, the average edge length of the MoS_2 domains will decrease and the thickness will increase, due to the greatly decreased Na content on the surface of glass.

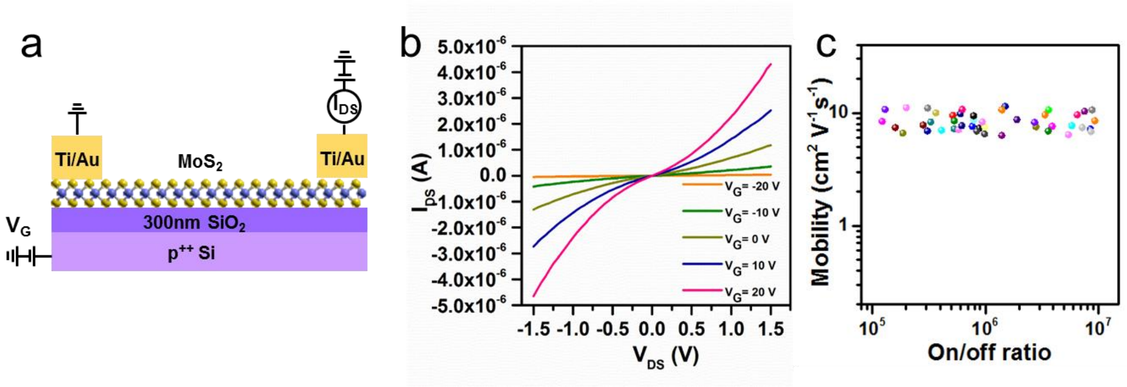

Supplementary Figure 13. Electrical properties of MoS_2-based FET devices. (a) The schematic diagram of the device. (b) I_{DS}-V_{DS} curves for a typical MoS_2 device. (c) A summary of the carrier mobility and the corresponding on/off ratio of 46 MoS_2 FET devices.

CVD-grown monolayer MoS_2	Mobility ($cm^2 V^{-1} s^{-1}$)	ON/OFF ratio	Method	Metal electrodes	Channel length and width	Pretreatment	Ref.
MoS_2/SiO_2	0.02	10^4	photolithography	Au	L=14 μm, W=60 μm	___	*Adv. Mater.*, **24**, 2320−2325 (2012)
MoS_2/SiO_2	0.2−3	10^4–10^6	EBL	5 nm Ti/50 nm Au	L=1 μm,	unannealed	*ACS Nano*, ***8***, 5304−5314 (2014)
MoS_2/SiO_2	1.2	10^7	EBL	5 nm Ti/50 nm Au	L=1 μm	___	Nano Lett. **13**, 1852−1857 (2013)
MoS_2/SiO_2	1−8	10^5–10^7	EBL	50 nm Al/5 nm Cr/50 nm Au	___	unannealed	Nat. Mater. **12**, 554–561 (2013)
MoS_2/SiO_2	10	10^6	Lithography and reaction ion etching	3 nm Ti/50 nm Au	L=100 μm, W=10 μm	___	Nat. Mater. **12**, 754–9 (2013)
MoS_2/glass	6.3−11.4	10^5–10^7	EBL	10 nm Ti/50 nm Au	L=1 μm, W=3 μm	unannealed	This work
MoS_2/SiO_2	8.2−11.4	10^6	EBL	1 nm Cr/30 nm Au	___	annealed at 150 °C for 1h	Nat. Commun. **6**, 6128 (2015)
MoS_2/glass	3−15	10^6–10^8	EBL	15 nm Cr/50 nm Au	L=8 μm, W=3 μm	unannealed	*Chem. Mater.*, **29**, 6095–6103 (2017)
MoS_2/SiO_2	64	10^7	EBL and oxygen plasma etching	30 nm Ni/20 nm Au	L=1,2,4,8,30 μm, W=4 μm	unannealed	*Adv. Funct. Mater.*, 1605896 **(**2017**)**
MoS_2/sapphire	90	10^7	UV-photolithography	5 nm Ti/30 nm Au	L=20 μm, W=10.7 μm	annealed at 450 °C for 4h	J. Am. Chem. Soc. **137**, 15632–15635 (2015).

Supplementary Table 1. Comparison of the electronic properties of back-gated FET devices fabricated with CVD-grown monolayer MoS_2 samples (measured at room temperature).

Supplementary References

1. Ju, M. *et al*. Universal substrate-trapping strategy to grow strictly monolayer transition metal dichalcogenides crystals. *Chem. Mater.* **29**, 6095–6103 (2017).

2. Zhang, Y. *et al.* Controlled growth of high-quality monolayer WS_2 layers on sapphire and imaging its grain boundary. *ACS Nano.* **7,** 8963–8971 (2013).

3. Chen, W. *et al*. Oxygen-assisted chemical vapor deposition growth of large single-crystal and high-quality monolayer MoS_2. *J. Am. Chem. Soc.* **137,** 15632–15635 (2015).
